# Convergent Evolution in a Murine Intestinal Parasite Rapidly Created the TGM Family of Molecular Mimics to Suppress the Host Immune Response

**DOI:** 10.1093/gbe/evad158

**Published:** 2023-08-25

**Authors:** Rick M Maizels, Stuart J Newfeld

**Affiliations:** Wellcome Centre for Integrative Parasitology, School of Infection and Immunity, University of Glasgow, Glasgow, United Kingdom; School of Life Sciences, Arizona State University, Tempe, Arizona, USA

**Keywords:** exon modularity, new gene origination, gene family neofunctionalization

## Abstract

The Transforming Growth Factor-β mimic (TGM) multigene family was recently discovered in the murine intestinal parasite *Heligmosomoides polygyrus*. This family was shaped by an atypical set of organismal and molecular evolutionary mechanisms along its path through the adaptive landscape. The relevant mechanisms are mimicry, convergence, exon modularity, new gene origination, and gene family neofunctionalization. We begin this review with a description of the TGM family and then address two evolutionary questions: “Why were TGM proteins needed for parasite survival” and “when did the TGM family originate”? For the former, we provide a likely answer, and for the latter, we identify multiple TGM building blocks in the ruminant intestinal parasite *Haemonchus contortus.* We close by identifying avenues for future investigation: new biochemical data to assign functions to more family members as well as new sequenced genomes in the Trichostrongyloidea superfamily and the *Heligmosomoides* genus to clarify TGM origins and expansion. Continued study of TGM proteins will generate increased knowledge of Transforming Growth Factor-β signaling, host–parasite interactions, and metazoan evolutionary mechanisms.

**Significance**

In this review, we describe the Transforming Growth Factor-β mimic (TGM) multigene family recently discovered in the murine intestinal parasite *Heligmosomoides polygyrus*. We also address two evolutionary questions: “Why were TGM proteins needed for parasite survival” and “when did the TGM family originate”? For the former, we suggest a need arising from atypical characteristics of the parasite's endogenous Transforming Growth Factor-β pathways. For the latter, we identify multiple TGM building blocks, but no intact TGM proteins, in the ruminant intestinal worm *Haemonchus contortus.* Our goal is to entice the evolutionary biology community to take a closer look at the creative solutions crafted by mammalian intestinal parasites to the selective pressure of the host immune response.

## Introduction

The Transforming Growth Factor-β mimic (TGM) family is a novel set of proteins expressed by a parasitic worm. This family was impacted by multiple organismal and molecular evolutionary mechanisms not normally seen together. The organismal mechanisms are mimicry and convergence. The molecular mechanisms are exon modularity, new gene origination, and gene family neofunctionalization. This review begins with a recap of each evolutionary process followed by an introduction to Transforming Growth Factor-β (TGF-β) signaling. We then offer summaries of parasite–host interactions, the parasite's TGF-β signaling pathway, and the structurally distinct TGM family that can suppress the host immune response. Next, we address two questions about TGM evolution: Why was a new gene family necessary and can we identify its origin and historical trajectory? Finally, we discuss TGM proteins in the context of the five mechanisms to illustrate how this family advances our understanding of each and to identify avenues for future investigation.

The TGM family was discovered in the murine intestinal roundworm *Heligmosomoides polygyrus* roughly 5 years ago (Johnston et al. 2017). The family was immediately noted as unusual due to its ability to mimic the immune regulatory functions of the host's own TGF-β proteins. The TGM family fits well within the concept that parasites will often find unique solutions to the strong selective pressure imposed by the host immune system. Evidence of this fit is that the TGM family of parasite-specific effector proteins has evolved so rapidly as to be considered lineage-specific (e.g., Jackson 2015).

Here, we place the TGM family into a larger evolutionary context. We start with an overview of the organismal and molecular mechanisms that impacted the TGM family and then summarize the TGF-β intercellular signaling pathway targeted by TGM proteins.

Mimicry has two variations. Müllerian mimicry is a protective mechanism utilized by two well-defended species, perhaps foul-tasting or venomous, that mimic each other's warning coloration to their mutual benefit (Muller 1878). Where mimics are not strongly protected by venom or other defenses, honest Müllerian mimicry becomes the better-known bluffing of Batesian mimicry (Bates 1862). In this protective mechanism, a defenseless species has adopted the warning coloration of a well-defended species. This benefits the defenseless but can dilute the protective value of the warning for the original species.

At the molecular level, mimicry is more often associated with offense than defense, such as when deployed by viruses (e.g., Albarnaz et al. 2022). Within intercellular signaling, mimicry is known but rare and typically function as antagonists. Examples of signaling receptor mimics are found in the Wnt and TGF-β pathways (described below). These mimics are able to bind ligands but cannot signal downstream. Two differences between TGMs and the known signaling mimics are that the TGM proteins are mimics of signaling ligands not receptors and that the TGM proteins characterized to date function as agonists not antagonists.

The mammalian TGF-β family of 33 secreted signaling proteins is divided into the TGF-β, Activin, and Bone Morphogenetic Protein (BMP) subfamilies (Wisotzkey and Newfeld 2020). Each TGF-β family member encodes a protein approximating the canonical format. There is an amino-terminal signal sequence, a 300-residue prodomain that is cleaved prior to secretion and then contributes to regulation, and a 110-residue ligand that binds to cell surface receptors. The ligand typically contains six cysteines that form three disulfide bonds creating a compact cystine knot structure (Hinck et al. 2016).

All TGF-β ligands form dimers that initiate signal transduction in a responsive cell by binding to a heterotetrameric complex of two type I and two type II receptors, both are transmembrane kinases. Type II receptors are constitutive kinases that phosphorylate type I receptors to activate them. Activated type I receptors then phosphorylate a receptor dedicated-Smad (R-Smad). Members of the multifunctional Smad family are committed to the intracellular signal transduction of TGF-β pathways. Once activated, a phospho-R-Smad binds to the Co-Smad, and the Smad complex translocates into the nucleus. In the nucleus, the Smad complex modulates the expression of target genes, typically in conjunction with cell-type specific transcription factors (e.g., Tzavlaki and Moustakas 2020).

One example of a signaling mimic is Bambi, an antagonist of BMP signaling. Bambi proteins are transmembrane mimics of the BMP type I receptor that lack an intracellular kinase domain. Bambi can form heterodimers with the BMP type I receptor. These heterodimers can bind to BMP type II receptor homodimers, but Bambi prevents the single type I receptor from activating signal transduction (Onichtchouk et al. 1999). A second example is Sizzled, an antagonist of signaling by Wnt proteins. Wnt ligands transmit information via a family of transmembrane receptors called Frizzleds. Sizzled proteins are secreted mimics of Frizzled receptors. Sizzled contains the extracellular Wnt-binding domain of Frizzled, but not the transmembrane or intracellular signal transducing segments. Sizzleds bind to Wnt proteins in the extracellular space and prevent them from binding to Frizzled receptors (Collavin and Kirschner 2003).

Convergent evolution is the mechanism that generates structures in different species that have similar function but were not present in their last common ancestor. The panda's thumb is a famous example, as opposable thumbs for grasping are associated with primates. The opposable thumbs of giant pandas are completely different in structure. Pandas have six fingers including the thumb that develops from a wrist bone entirely separate from the other fingers (Endo et al. 1999).

At the molecular level, an example of the functional convergence of dissimilar molecules is the adaptive immune system of the lamprey. These jawless vertebrates use two lymphocyte receptors that contain variable numbers of leucine-rich repeat segments as antibodies, in contrast to the immunoglobulin-based antibodies of jawed vertebrates. Instead of a recombination between immunoglobulin chains creating antibody diversity, in lamprey antibody diversity is achieved via multiple insertions of leucine repeats into the pair of receptor genes (Guo et al. 2009).

Exon modularity was proposed as a mechanism for new gene formation that explained the existence of chimeric genes (Gilbert 1978). These were outside the scope of the original model for new gene formation by duplication (Muller 1936). An example of a new modular gene is *jingwei*, a gene found in only two species of African Drosophila (Long and Langley 1993). *jingwei* was created by illegitimate recombination that merged parts of two existing genes (*Adh* and *ymp*) into a hybrid gene. Subsequent sequence evolution driven by positive selection rapidly endowed *jingwei* with functions distinct from either parent. In this mechanism, exons are functional modules that can be stitched together from unrelated genes to create a new gene (Long et al. 1995).

New gene origination occurs via multiple mechanisms. In humans and flies, the classical mechanism of duplication is estimated to be the most common at roughly 80%, followed by retrotransposition via mobile elements at 10%, and de novo formation from previously noncoding DNA at roughly 5%. All other mechanisms together, such as whole genome duplication and modular generation, yield the remaining 5% (Chen et al. 2013; Long et al. 2013). The 33-member TGF-β family in mammals is an example of repeated gene duplication accomplished by copy and paste of genomic DNA from one region to another that maintains, at least initially, the original gene's intron/exon organization (e.g., Newfeld et al. 1999). The mouse paralogs Arxes1 and Arxes2 are intronless copies of an ancestral gene (Spcs3) and an example of retrotransposition, the copy and paste of genomic DNA from one region to another via an mRNA intermediate that eliminates introns (e.g., Prokesch et al. 2011). The examples of de novo new gene formation are the low-complexity antifreeze proteins of polar fish created from the previously noncoding microsatellite DNA (Chen et al. 1997).

Gene family neofunctionalization is a mechanism for diversification of protein function. Initially, a new gene created by duplication retains the original function. Over time, either the new gene or the original gene acquires mutations that provide a distinct yet beneficial function. This new function is then preserved by natural selection. An example is the four-member *Sdic* gene family that exists only in *Drosophila melanogaster*. This family originated by duplication from *Cdic* then acquired two new features: conversion of an intron into an exon, and conversion of a different intron into a testis-specific promoter (Nurminsky et al. 1998).

In the next section, we provide a summary of interactions between the intestinal parasite *H. polygyrus* and the mammalian host's TGF-β signaling pathway. Then, we provide a brief description of TGF-β pathway components in *H. polygyrus*. This is followed by a detailed review of the structure and sequence of the *H. polygyrus* family of ten TGMs. Next, we address two questions about TGM evolution: with its own TGF-β signaling pathway, why was a new gene family of TGMs necessary for parasite survival and can we identify counterparts of the TGM family in other species to suggest its origin and trace its historical trajectory in *H. polygyrus*? We end by discussing TGM proteins in the context of the five evolutionary mechanisms and identifying two avenues for future investigation.

### Parasites and TGF-β Signaling in Their Host

Parasites, both protozoan and metazoan, are able to successfully overcome the immune defenses of their host species to establish long-term infections. They target multiple pathways of the immune system through a myriad of secreted modulators and effectors that block the activation, differentiation, and mobilization of immune cells (Maizels et al. 2018). The cytokine TGF-β is a major regulator of immune responses, controlling reactions to both endogenous (self-) and exogenous (nonself) specificities (Li et al. 2006). As a result, in many parasite infections, TGF-β signaling is associated with expansion of immunosuppressive regulatory T-cells (T-regs) that inhibit host antiparasite activity (Johnston et al. 2016; Maizels 2021).

In most cases, pathogens manipulate host responses to favor production or activation of TGF-β at the site of infection. However, it is emerging that some species secrete proteins that directly interact with host TGF-β receptors without the involvement of the host ligand. This notion is particularly applied to worm parasites related to the free-living model nematode *Caenorhabditis elegans*. This species encodes five members of the TGF-β gene family as well as type I and II receptors. Among its TGF-β ligands, abnormal DAuer Formation (DAF)-7 has the highest similarity to mammalian TGF-β, and its expression promotes the development of the larval stage (Ren et al. 1996).

A DAF-7 homolog was found in *Brugia malayi*, a human parasite that causes lymphatic filariasis. The DAF-7 homolog was maximally expressed in blood-stage microfilaria that is associated with immune hyporesponsiveness in infected individuals (Maizels and Lawrence 1991). When this homolog was expressed in insect cells, culture supernatants were able to activate a mammalian TGF-β responsive cell line (Gomez-Escobar et al. 2000). In subsequent studies, TGF-β homologs from two species of trematodes (phylogenetically very distant from nematodes) were described; TGF-Like Molecule from the flatworm *Fasciola hepatica* bound to mammalian TGF-β receptors, although its impact was limited (Sulaiman et al. 2016). An Activin homolog in the tapeworm *Echinococcus multilocularis* was active in mammalian cells, but only in the presence of TGF-β (Nono et al. 2020).

Extensive data are derived from the studies of *H. polygyrus*, a roundworm intestinal parasite that establishes chronic infections in strains of laboratory mice. Infected mice show an expansion of T-regs, and they can be rendered more susceptible or resistant to parasite infection by depleting or increasing the T-reg population (Smith et al. 2016). In addition, a pharmacological inhibitor of TGF-β receptors (SB431542; GlaxoSmithKline) when administered to infected mice resulted in a reduction of parasite burden. Following the hypothesis that the parasite is actively inducing T-reg differentiation, excretory/secretory (ES) products released from parasites cultured in vitro were screened for TGF-β activity (Grainger et al. 2010). Parasite ES products induced Smad phosphorylation in the murine embryonic fibroblast cell line MFB-F11, which cannot produce endogenous TGF-β (Tesseur et al. 2006). In addition, signaling was blocked only by SB431542 and not by antibodies to mammalian TGF-β. Most strikingly, in T-cells, ES products could induce the expression of the T-reg–specific transcription factor Foxp3 to an equal extent as TGF-β, confirming that an endogenous component from the parasite can redirect the murine immune system toward an immunosuppressive phenotype.

**Fig. 1.**
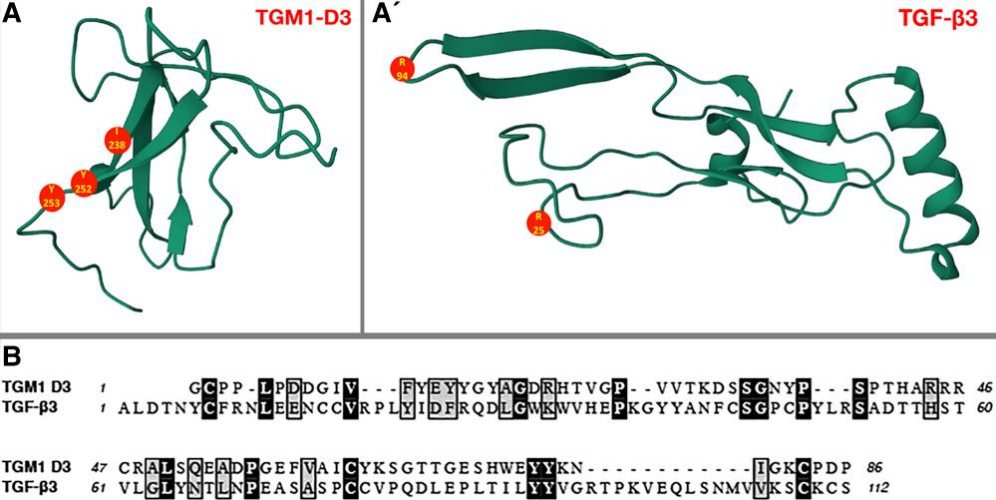
TGM1 D3 shares little structural or sequence similarity with TGF-β3. (*A*, *A*ʹ) Little similarity between the structures of *H. polygyrus* TGM1 D3 (PDB: 7SXB or AlphaFold A0A2D1LW19) and a monomer of the ligand domain of human TGF-β3 (PDB: 1TGK). The type II receptor binding face is oriented to the left for each with their β-strands indicated as flat arrows. Critical amino acids facilitating receptor binding are in red as determined by mutational analysis for TGM D3 (Mukundan et al. 2022) or the crystal structure of the ligand/receptor complex for TGF-β3. (*B*) Alignment of *H. polygyrus* TGM1 D3 (ATO59092 residues 177–262) and the ligand domain of human TGF-β3 (P10600, residues 301–412). There is also little sequence similarity: 13.4% (15/112) identity in black and 24.1% (27/112) identical plus similar in black and gray.

**Fig. 2.**
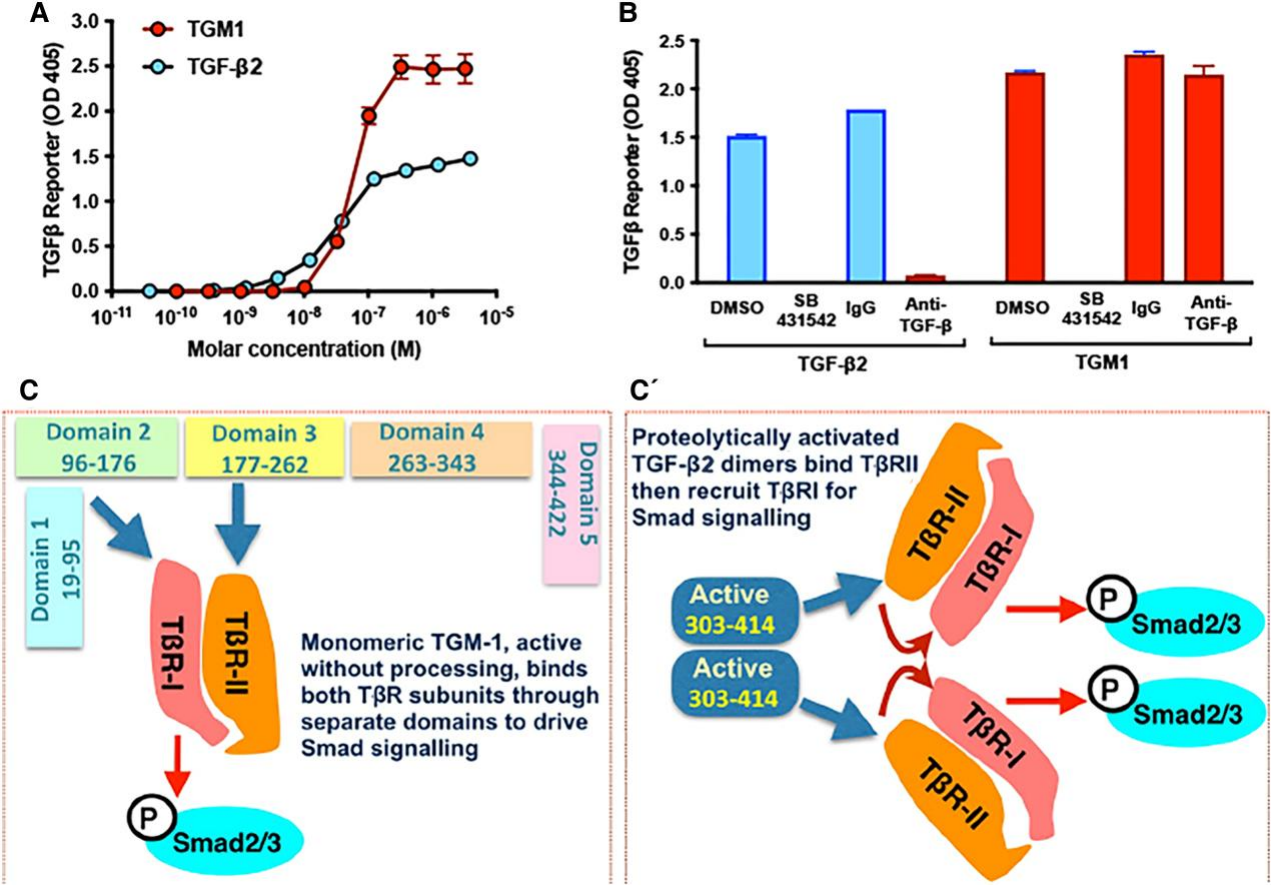
The TGM1 domains 1–3 activate TGF-β receptors via a mechanism distinct from TGF-β2. (*A*) TGM1 activates TGF-β responses more potently than exogenous human TGF-β2 in MFB-F11 reporter fibroblast cell line. This line lacks endogenous TGF-β2 and contains a Smad-inducible secreted alkaline phosphatase (redrawn from Johnston et al. 2017). (*B*) Responses to both TGF-β2 and TGM1 are abolished by the ALK5 kinase inhibitor SB431542. Only TGF-β2 responses are blocked by a pan-TGF-β antibody; control assays employed DMSO or normal mouse IgG (redrawn from Johnston et al. 2017). (*C*, *C*ʹ) Schematic representation of the distinct mechanisms employed by the TGM D1–3 and TGF-β2 ligands. Shown are the interactions of D1–-3 of TGM1 (*C*) with type I and type II receptors in comparison with the interactions of ligand dimers of TGF-β2 (*C*ʹ) with only the type II receptor. In both cases, Smads are phosphorylated, and downstream signals are propagated.

As *H. polygyrus* has at least one TGF-β family member (DAF-7; McSorley et al. 2010), it was expected that this protein was responsible for mammalian TGF-β receptor interactions. However, this was not the case. Instead, when proteins secreted by this parasite were biochemically fractionated and screened for TGF-β activity, a novel product with no sequence or structural similarity to the TGF-β family (fig. 1) was identified (Johnston et al. 2017). The new *H. polygyrus* protein potently activated signaling through the TGF-β receptors and downstream Smad mediators (fig. 2). Binding between recombinant parasite protein and the type I and type II TGF-β receptors was confirmed by surface plasmon resonance (Johnston et al. 2017). Hence, the new protein was designated TGF-β Mimic1 (TGM1).

In contrast to TGF-β that requires dimerization, proteolytic cleavage of signal peptides and prodomains and then release of the ligand dimer from their prodomains for receptor binding (Annes et al. 2003), TGM1 needs no processing beyond removal of its signal peptide. TGM1 is readily produced as an active recombinant protein that activates the MFB-F11 TGF-β reporter cell line at a higher level than TGF-β itself (fig. 2). Recombinant TGM1 can drive both murine and human T-cells into the suppressive T-reg phenotype to a greater degree than TGF-β, as measured by an induction of Foxp3 expression (Cook et al. 2021; White et al. 2021). When administered in mouse models, TGM1 significantly dampened airway allergic inflammation (Chauché et al. 2022) and intestinal colitis (Smyth et al. 2021, 2023), as well as prolonged the survival of fully allogeneic skin grafts (Johnston et al. 2017). Hence, TGM1 is a potent immunosuppressor that acts by expansion of regulatory T-cells.

### TGM Structure and Gene Family

**Fig. 3.**
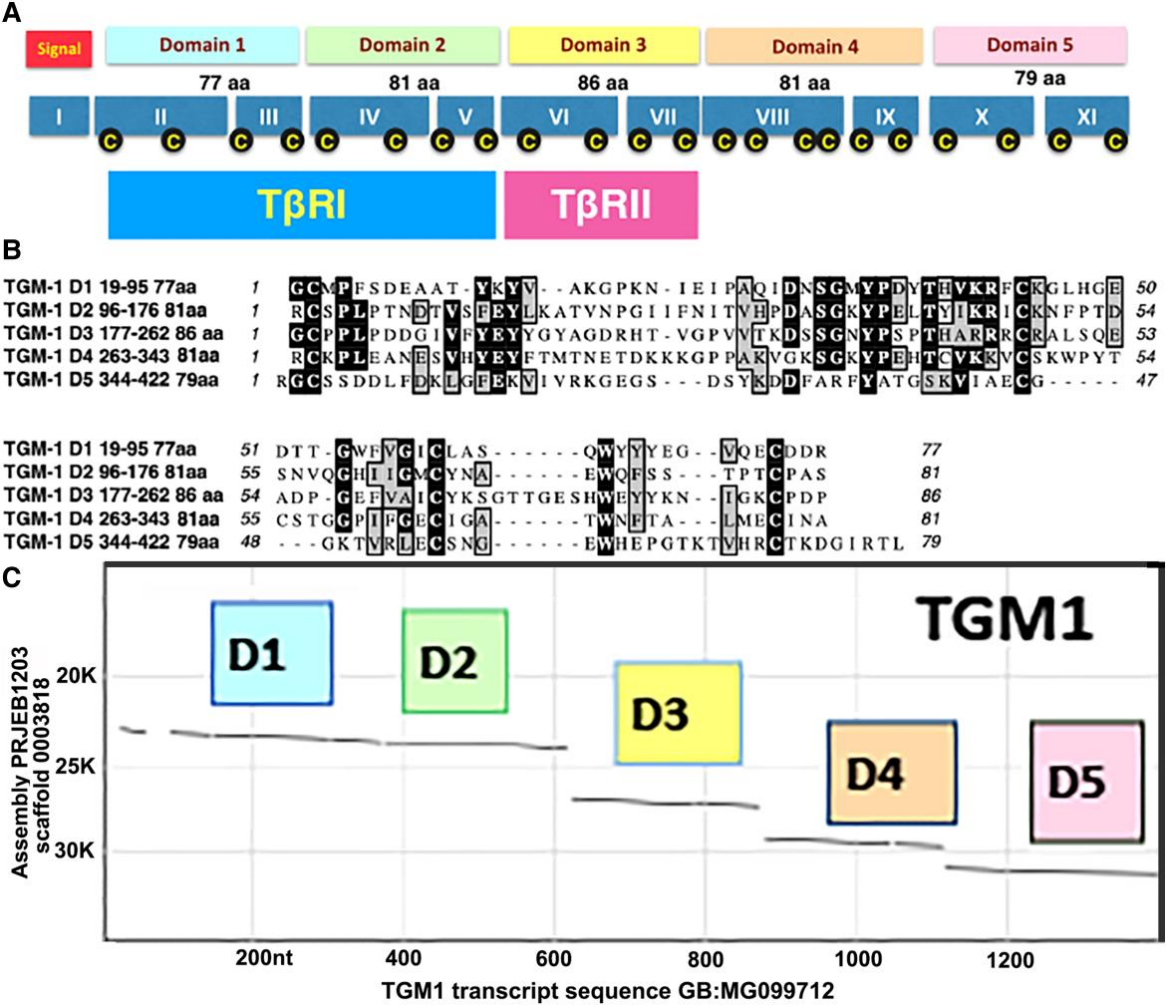
TGM1 domain organization, alignment, and introns. (*A*) Top row displays the five domains of TGM1 in different colors with their amino acid length. The middle row of teal-shaded boxes represents exons 1–11 in roman numerals. Each domain is encoded by two exons, each with a pair of cysteines, apart from exon VIII with four cysteines. The bottom row of rectangles indicates the distinct functions of D1/2 and D3 in binding to a different type of TGF-β receptor. (*B*) Amino acid alignment of the five domains of TGM1 with identities shown in black and similarities in gray. The four cysteines plus a tyrosine (Y; top row) and a tryptophan (W; bottom row) are invariant. (*C*) Dot plot of the TGM1 mRNA sequence against its genomic scaffold revealing intron sizes. The intradomain introns are only 50–70 nucleotides (barely visible as discontinuities in each domain). The interdomain introns are roughly 20-fold larger at 1–3 kb.

Although TGM has a novel sequence, a distant similarity to the ancient Complement Control Protein (CCP) or Sushi domain was identified (Johnston et al. 2017). Within the full-length TGM1 protein are five similar but nonidentical CCP domains. Each domain is roughly 80 amino acids in length and contains either 4 or 6 cysteines that form intrachain disulfide bonds. Each domain is encoded by two exons with intradomain introns being much shorter than interdomain introns (fig. 3). The assignment of a specific function to each domain was based on empirical data obtained with recombinant expression of individual domains in soluble form. Of the five domains, domains 1 and 2 (D1/2) are responsible for binding to the mammalian type I receptor TβRI at high affinity, whereas D3 binds to the type II receptor TβRII (Mukundan et al. 2022). The ability of a monomeric TGM1 protein to bind, via distinct domains, to TβRI and TβRII presents a contrast to mammalian TGF-β that binds as a dimer to TβRII, which only then is able to recruit TβRI into the signaling complex.

**Fig. 4.**
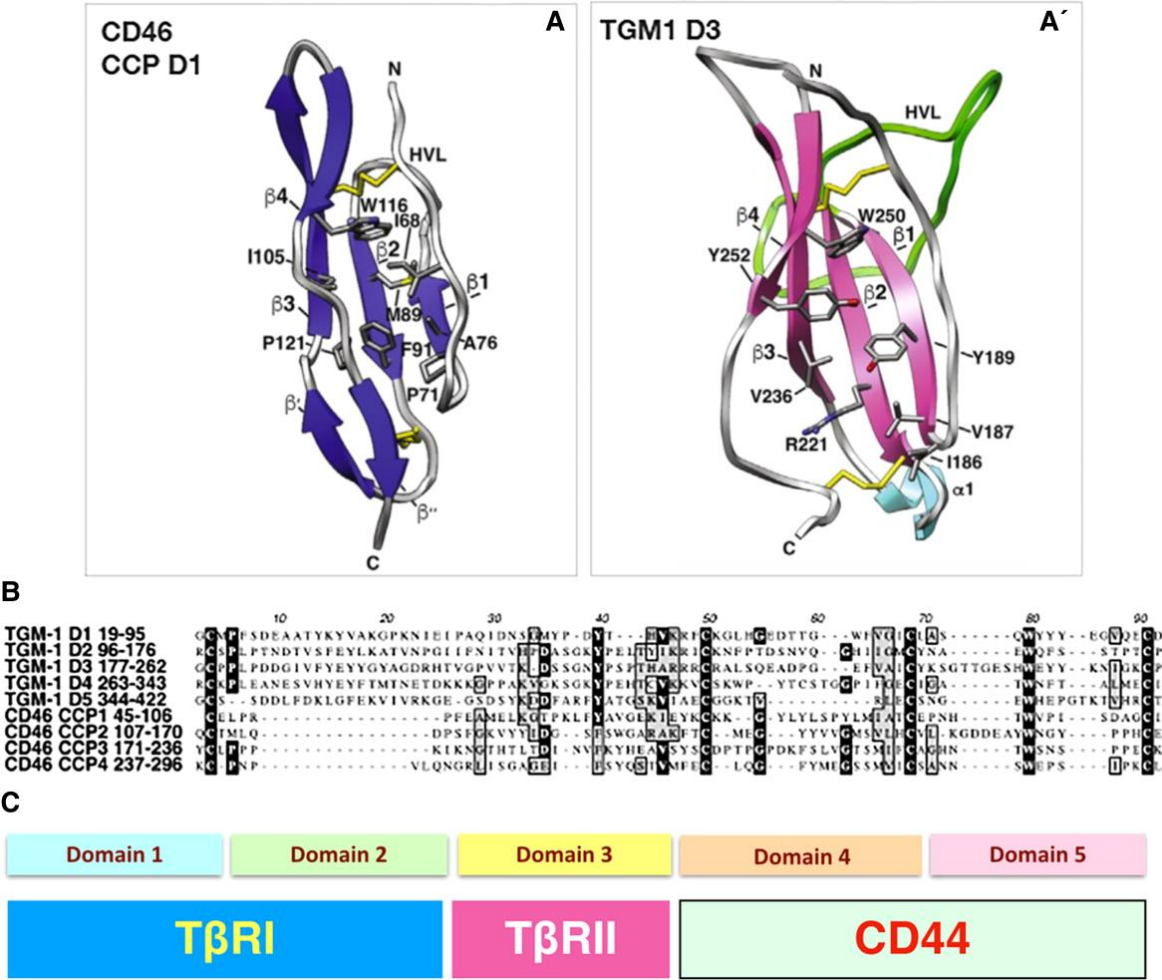
TGM1 D3 shares structural but not sequence similarity with a human CCP domain, and TGM1 D4/5 bind to the immune cell surface protein CD44. (*A*, *A*ʹ) Similar structures of CCP domain 1 from the human membrane cofactor protein CD46 (PDB: 1CKL) and *H. polygyrus* TGM1 D3 (7SXB; reproduced from Mukundan et al. 2022). In each panel, β-strands are shown as flat arrows; the two pairs of disulfide bonded cysteines (one pair near the top and the other near the bottom) are yellow with additional residues as indicated. (*B*) Alignment of *H. polygyrus* TGM1 D1–5 with human CD46 CCP1-CCP4 revealing little sequence similarity beyond the four cysteines. Comparing CD46 CCP1–CCP4 with TGM1 D3, there is 9.5% (8/84) identity and 19.1% (16/84) identity plus similarity. The alignment is artificially truncated at the first and last cysteine to avoid ungainly extensions. (*C*) Rectangles below the domains of TGM1 reflect their function, with D4/5 recently shown to bind to the immune cell coreceptor CD44 (van Dinther et al. 2023).

In addition to TGM1, two other proteins containing multiple CCP domains are prominent in the secretome of *H. polygyrus* (Hewitson et al. 2013). These are immunomodulators that bind to the cytokine IL-33 (Alarmin Release Inhibitor; Osbourn et al. 2017) or the IL-33 receptor (Binds Alarmin Receptor and Inhibits; Vacca et al. 2020). As the sequence similarity between these three proteins is low, they suggest flexibility in the structure of a CCP domain. Such versatility is also apparent in the recently resolved structure of TGM1 D3 (fig. 4). The structure reveals the loss of two β-strands and the insertion of a 23-residue loop relative to canonical CCP structures from mammalian proteins. Remodeling creates a hydrophobic surface for receptor interaction that allows D3 to bind to the same residues of TβRII that are contacted by TGF-β despite the two ligands differing radically in overall structure (Mukundan et al. 2022).

**Fig. 5.**
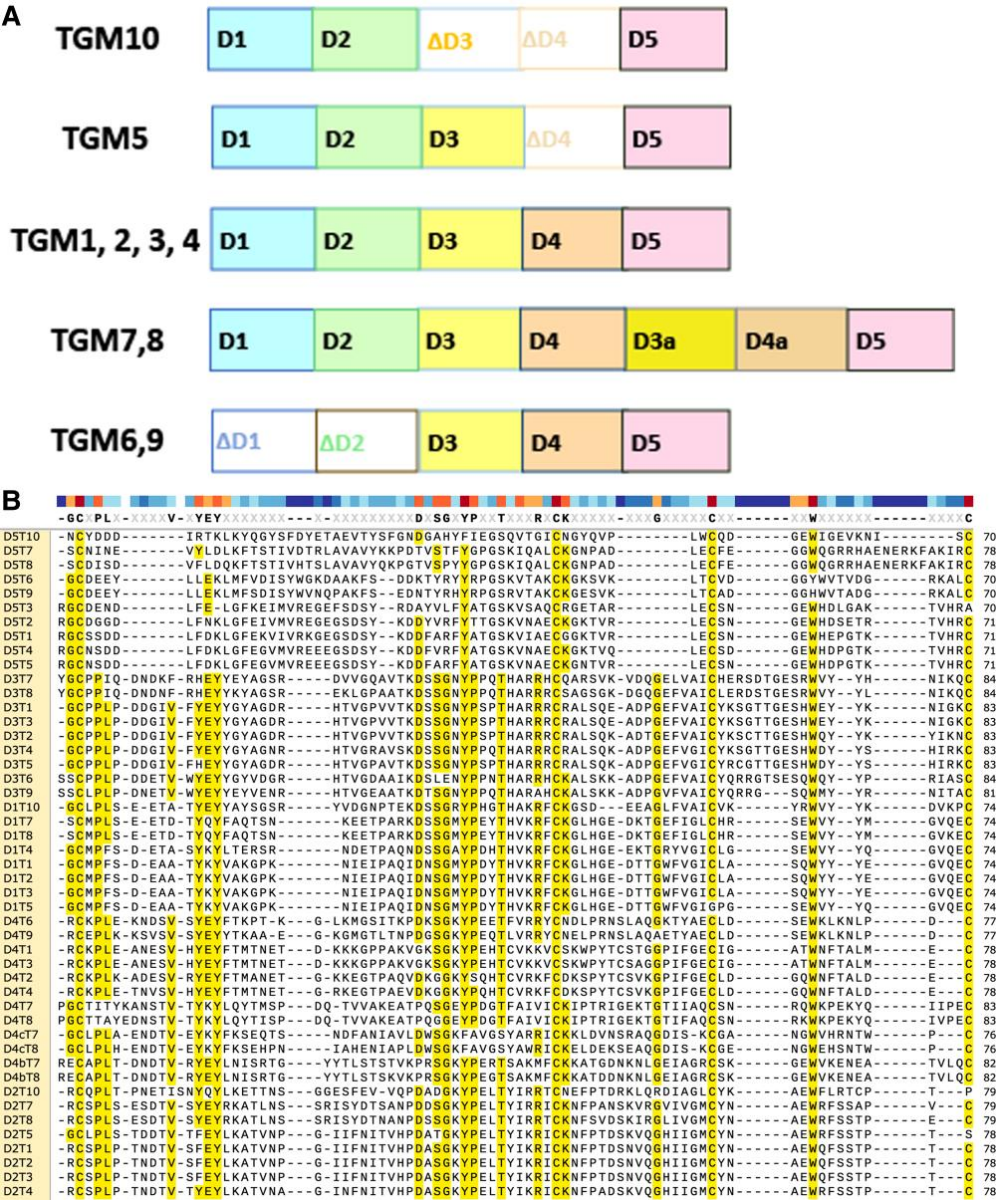
TGM family diversification of domain number and sequences. (*A*) Schematic shows that TGM family members experienced repeated gain of new domains. TGM10 with only three domains appears to be the oldest, and thus, the absence of D3/4 may not be a loss. The loss of D1/2 in TGM6,9 may have occurred contemporaneously with the gain of D3*a*/4*a* in TGM7/9 (redrawn from Smyth et al. 2018). (*B*) Alignment of all domains from all TGM family members. This was created in Clustal Omega with default parameters (www.ebi.ac.uk/Tools/msa/clustalo/). The alignment ends at the final conserved C in each domain to eliminate variation in extensions. An amino acid present in more than 50% of domains is highlighted in yellow. A consensus sequence of the residues with more than 50% conservation is shown above the alignment. Above the consensus is a color-coded rendition of that sequence with red (100%) and orange (>50%) indicating the highest levels of conservation. Three shades of blue from light to dark indicate an increasing number of domains contain a gap. White signifies that only one or two domains have an amino acid at that position.

Further proteomic analyses of the secretome of *H. polygyrus* identified additional proteins related to TGM (Smyth et al. 2018). There are five additional proteins from adults that inhabit the intestinal lumen and four from larvae that encyst in intestinal muscle (fig. 5). These proteins, designated TGM1–6 from the adult (TGM1 being the original) and TGM7–10 from larvae, possess between three and seven tandemly repeated but nonidentical CCP domains, all more closely related to each other than to mammalian CCP sequences. Each domain of a single TGM protein has a unique identity, and only D5 is universally shared by all ten proteins. Those containing the same five domains as TGM1 are the other adult expressed proteins TGM2/3/4, although only TGM1/2/3 are able to activate signaling in the TGF-β reporter cells (Smyth et al. 2018). Although TGM5 is missing D4, other TGM proteins lack domains that mediate receptor binding in TGM1. These are TGM6,9 (D1/2 absent) and TGM10 (D3/4 absent). Conversely, two larval proteins (TGM7,8) have acquired additional domains through an apparent duplication of D3/4. At this time, receptors or coreceptors for many of these domains remain unidentified.

The domains of the TGM family likely grew from an ancient CCP module by repeated duplication. Coincident convergent evolution has conferred on D1–3 the capacity to interact with both TGF-β receptors. Recently, the role of D4/5 in TGM1 was reported as binding to the CD44 coreceptor expressed on the surface of most immune cells (fig. 4). Functionally, D4/5 binding to the CD44 coreceptor enhances the potency of TGM1 signaling. This is shown by the diminished induction of T-regs when a truncated form of TGM1 (D1–3 only) is added to T-cells (van Dinther et al. 2023). These results suggest that D4/5 fulfill a cell type-specific role in potentiating the TGM1 T-cell interactions. By extrapolation as D4/5 show the greatest amino acid diversity across TGM proteins, each TGM family member could recognize a different cell type.

### TGM Family Evolution

Two evolutionary questions about the ten-member TGM family of multidomain proteins in *H. polygyrus* are addressed here. First, why were TGM proteins needed for *H. polygyrus* survival? More precisely, why did a mouse intestinal parasite under selective pressure to evade the host immune system need convergent evolution of the ancient CCP domain to create mimics of immune modulatory TGF-β proteins? Second, when did the TGM family originate? More precisely, can TGM proteins or individual TGM domains be identified outside *H. polygyrus* or is the family limited to this specific host–parasite pair?

As context for the first question of why, note that the TGF-β family and its signaling pathway are present in all multicellular animals (Tzavlaki and Moustakas 2020). This includes *H. polygyrus* (McSorley et al. 2010) and all taxa containing parasitic nematodes (Viney et al. 2005). If present, then why were endogenous TGF-β proteins not weaponized by the parasite to modulate the host immune system? Employing existing proteins avoids the tedious and error-prone evolution of mimics by convergence. On the other hand, parasite life cycles create atypical circumstances, and perhaps *H. polygyrus* has repurposed its TGF-β proteins.

**Fig. 6.**
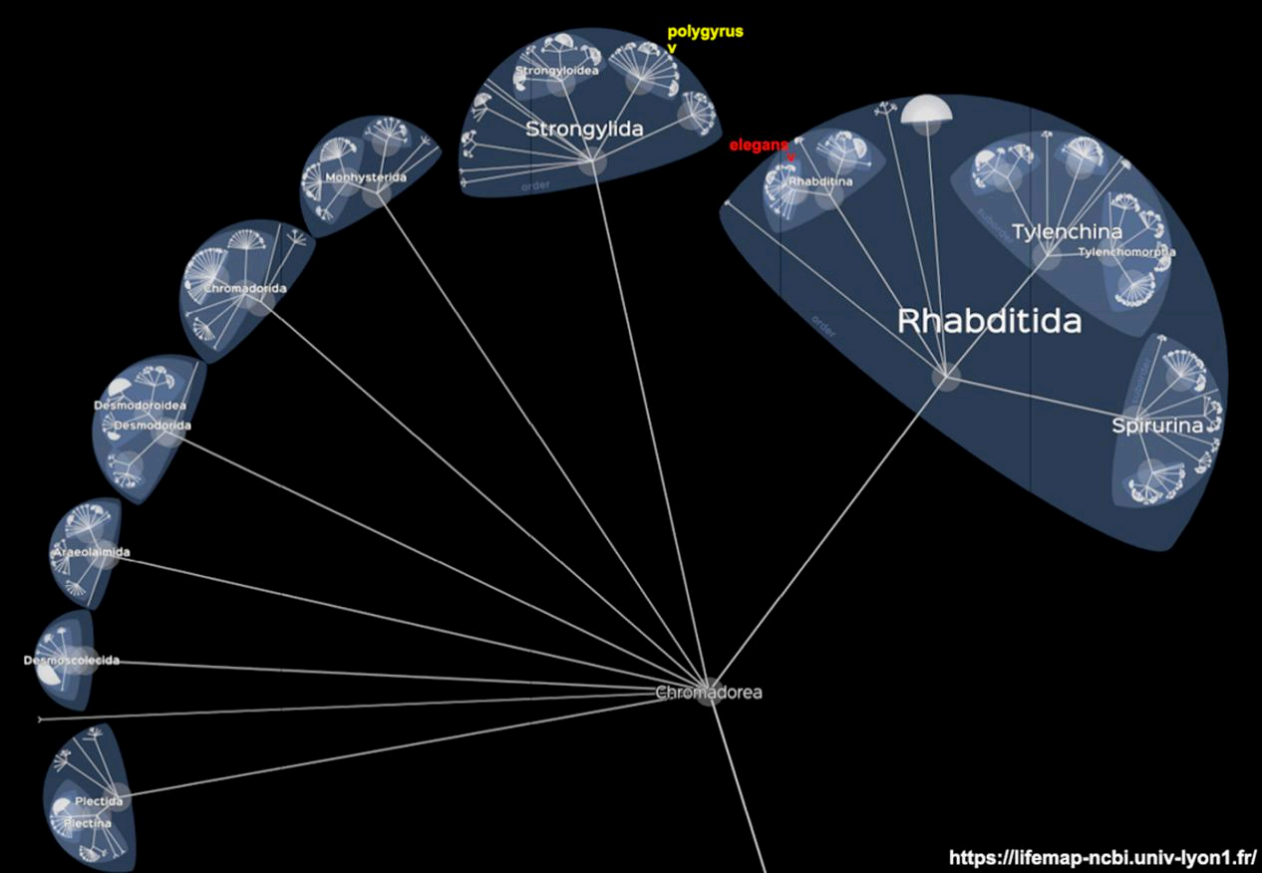
Taxonomy of the class Chromadorea of the phylum Nematoda. Shown is a screenshot from the NCBI version of Lifemap (de Vienne 2016; https://lifemap-ncbi.univ-lyon1.fr/). The location of the free-living soil model organism *C. elegans* in the order Rhabditida is indicated by a red arrowhead. The location of the murine intestinal parasite *H. polygyrus* in the order Strongylida is indicated by a yellow arrowhead. Unlike a phylogenetic tree, in this tree the proximity of the indicated species is artificial because the placement of the orders around the node is random. The length of the branches is purely illustrative and does not convey any evolutionary distance information. The divergence estimate for Chromadorea (table 1) is from timetree.org that relies on published data to estimate the age of the last common ancestor.

Taxonomically, *C. elegans* and *H. polygyrus* belong to the class Chromadorea within the phylum Nematoda. Their divergence is estimated at 541 Ma (fig. 6). For comparison, divergence within the subphylum Vertebrata of the lamprey and human is 599 Ma (timetree.org; [Kumar et al. 2022]). The five *C. elegans* TGF-β family members belong to all three subfamilies (Wisotzkey and Newfeld 2020). DAF-7 in the TGF-β subfamily functions via the type I DAF-1 and type II DAF-4 receptors (Patterson et al. 1997). DBL-1 in the BMP subfamily functions via the type I SMA-6 and type II DAF-4 receptors (Berg et al. 2019). UNC-129 in the TGF-β subfamily, TIG-2 in the BMP subfamily, and TIG-3 in the Activin subfamily function via the type I receptor SMA-6 alone (Baltaci et al. 2022).

To address the question of why, we searched for TGF-β family members in the *H. polygyrus* genome. We employed BLASTp, with the *C. elegans* proteins as queries and default parameters. We easily identified a sequence matching each of the five *C. elegans* TGF-β family members with high confidence (table 1). Four matches are in full length with an obvious furin cleavage site and all ligand cysteines. For TIG-3, the match is partial with only the last five ligand cysteines. There is a partial second match to *C. elegans* DAF-7 starting at the cleavage site that contains all ligand cysteines. The second hit matches *C. elegans* DAF-7 less well (35 identities across the 110-residue domain) than the initial match (59 identities in the same region). We conclude that *H. polygyrus* has a full complement of TGF-β family members. The question of why *H. polygyrus* did not weaponize them against the mouse immune system persists.

**Table 1:** Six Matching TGF-β Ligands (Chromadorea Divergence 541 Ma^a^)

*Caenorhabditis elegans* ^b^ (GenBank)	*Heligmosomoides polygyrus* ^c^ (GenBank)	BLASTp % Identity^d^ (Residues)
Dauer larva development regulatory growth factor DAF-7 (NP_497265)	Transforming growth protein 2-like protein partial (ACR27076)	32% (357) full length
Protein DBL-1 (NP_504709)	Unnamed protein product (VDO89536)	45% (326) full length
BMP-like protein UNC-129 (NP_501566)	Unnamed protein product (VDP24209)	43% (351) full length
TGF-β2 domain-containing protein (TIG-3; NP_497318)	Unnamed protein product (VDO68844)	30% (257) full length
TGF-β2 domain-containing protein (TIG-2; NP_504271)	Unnamed protein product (VDO82768)	54% (87) partial
	ACR27076 duplication unnamed protein product (VDP00442)	44% (165) ACR27076 34% (100) DAF-7

a Timetree.org (Kumar et al. 2022).

b Nematoda, Chromadorea, Rhabditida, Rhabditoidea, and Peloderinae (free living in soil).

c Nematoda, Chromadorea, Strongylida, Trichostrongyloidea, and Heligmosomatidae (intestinal parasite of mouse).

d For reference, meaningless match is 5%, as 1 out of 20 amino acids at random will match the query at any position.

Perhaps, *H. polygyrus* TGF-β proteins diverged sufficiently such that they would not bind to the mammalian TGF-β receptors and thus cannot be weaponized. To examine this possibility, we attempted to find *H. polygyrus* matches to the three *C. elegans* TGF-β receptor proteins. We noted that the *C. elegans* type II DAF-4 has five reference sequences. We scanned *H. polygyrus* with all five plus the DAF-1 and SMA-6 reference sequences employing tBLASTn at slightly reduced stringency to pick up both annotated and unannotated matches. To our surprise, all seven *C. elegans* receptor reference sequences identified the same sequence in *H. polygyrus* as their top match (table 2). The identified *H. polygyrus* sequence has an extracellular Cys box found in type I and type II receptors, an intracellular GS box found in type I receptors, a transmembrane domain, and an intracellular serine/threonine kinase domain found in both types of receptors (e.g., Brummel et al. 1994). In the sole *H. polygyrus* sequence, the Cys box is very close to the predicted initiator methionine, and there is no signal sequence (Signal6.0; [Teufel et al. 2022]). This structure suggested to us that the *H. polygyrus* sequence is incomplete. However, even at further reduced stringency, neither BLASTp nor tBLASTn identified any *H. polygyrus* sequences matching the ligand binding regions of any of the reference sequences for all *C. elegans* TGF-β receptors.

**Table 2:** Single Match to Type I and Type II TGF-β Receptors (Chromadorea)

*Caenorhabditis elegans* ^a^	*Heligmosomoides polygyrus* ^b,c^	tBLASTn % Identity (Residues)
Receptor protein serine/threonine kinase DAF-4 (NP_001021149; 446aa)	Unnamed protein product (VDP08015) alignment starts transmembrane region	24% (263) partial
Receptor protein serine/threonine kinase DAF-4 (NP_001317770; 525aa)	Unnamed protein product (VDP08015) alignment starts transmembrane region	28% (418) partial
Serine/threonine protein kinase receptor DAF-4 (NP_001379620; 592aa)	Unnamed protein product (VDP08015) alignment starts extracellular at Cys box	28% (418) partial
Serine/threonine protein kinase receptor DAF-4 (NP_001309588; 723aa)	Unnamed protein product (VDP08015) alignment starts transmembrane region	27% (444) partial
Cell surface receptor DAF-4 (NP_498211; type II; 744aa)	Unnamed protein product (VDP08015) alignment starts transmembrane region	28% (418) partial
Cell surface receptor DAF-1 (type I; NP_001023159)	Unnamed protein product (VDP08015) alignment starts extracellular at Cys box	36% (465) partial
Serine/threonine-protein kinase receptor SMA-6 (type I; NP_495271)	Unnamed protein product (VDP08015) alignment starts intracellular at GS box	35% (361) partial

a Note that DAF–4 has five references sequences of distinct lengths; only the two longest have a ligand binding domain.

b Match to full length; BLASTp and tBLASTn of ligand binding region failed even at reduced stringency.

c Match lacks an obligatory signal sequence for pushing the extracellular portion through the membrane and thus may be incomplete perhaps explaining the absence of a ligand binding region.

Taken together the receptor data, although circumstantial, suggests that the mechanism of *H. polygyrus* TGF-β signaling is sufficiently distinct from the standard mechanism that *H. polygyrus* ligands would not bind to mammalian TGF-β receptors. Thus, modulation of the host immune system with endogenous *H. polygyrus* TGF-β ligands may not be feasible, providing a potential “why” to the existence of TGM proteins. Selective pressure to evade the host immune response then led to TGMs via duplication and convergent evolution from a preexisting CCP module.

**Fig. 7.**
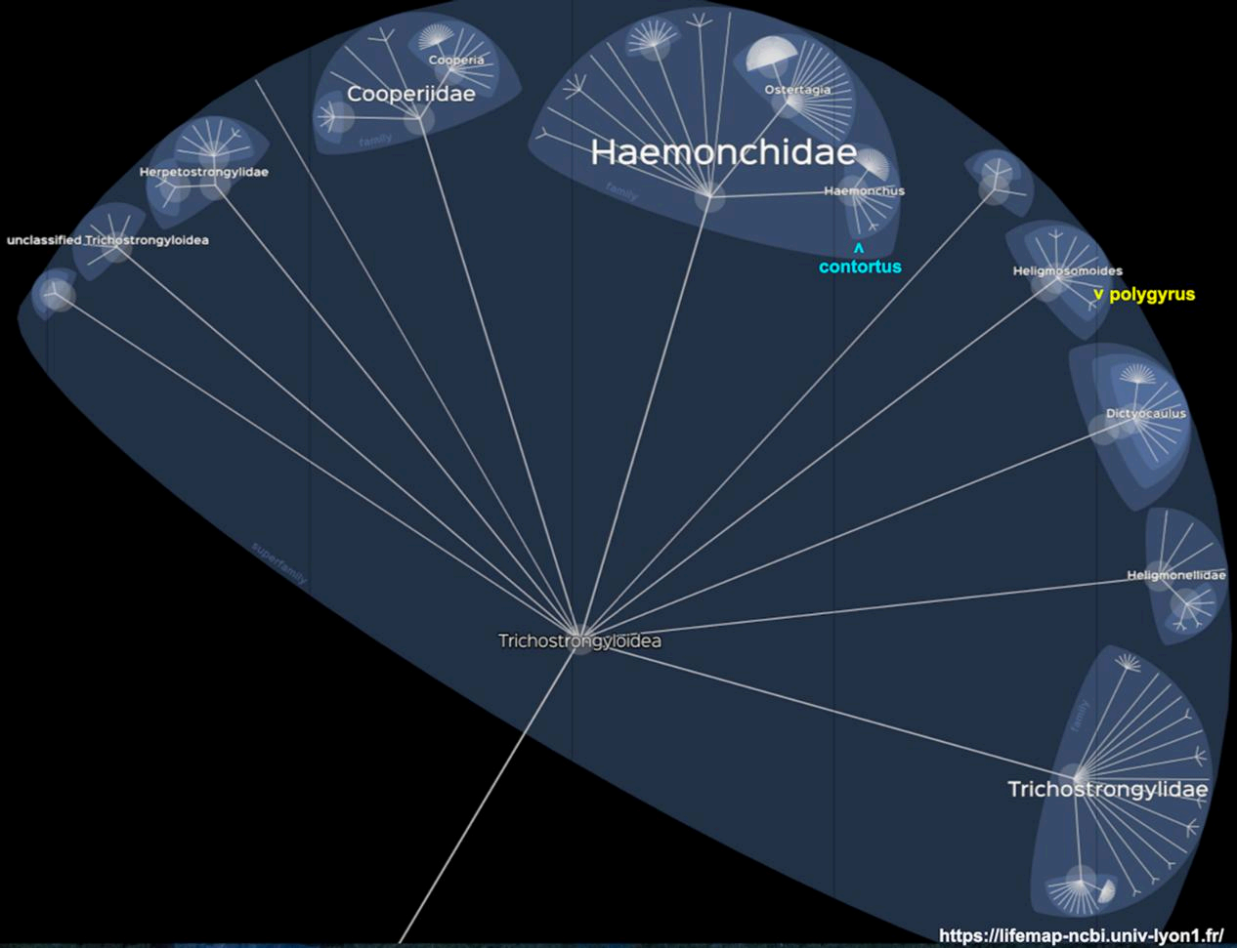
Taxonomy of the superfamily Trichostrongylidea in the order Strongylida. Shown is a screenshot from the NCBI version of Lifemap as described in figure 6. The location of the ruminant intestinal parasite *H. contortus* in the family Haemonchidae is indicated by a blue arrowhead. The location of *H. polygyrus* in the genus *Heligmosomoides* is indicated by a yellow arrowhead. The divergence estimate for Trichostrongylidae (table 2) is by analogy to the timetree.org divergence of its Chromadorea sister superfamily Rhabditoidea shown in figure 6.

As context for the question of when, note that in the superfamily Trichostrongyloidea in addition to *H. polygyrus*, only one species has a “chromosome” level assembly, *Haemonchus contortus* (fig. 7). This sequence derives from a laboratory-maintained strain at the Moredun Research Institute, UK (Laing et al. 2013, 2016). *Haemonchus contortus* shares the intestinal parasite lifestyle with *H. polygyrus* although it colonizes ruminants. The divergence time for Trichostrongyloidea is unknown. A simplistic analogy can be made to its sister superfamily Rhabditoidea that contains *C. elegans* with a divergence of 186 Ma. The divergence time for the worms’ murine and ruminant hosts in the Boreoeutheria (a magnorder within placental mammals) is 94 Ma. In other words, the common ancestor of *H. contortus* and *H. polygyrus* was likely present in the common ancestor of their hosts.

We checked the assemblies of the four “scaffold” level species for completeness by BLASTp with default parameters employing *C. elegans* TGF-β ligands and receptors. Although *H. contortus* has matches to multiple ligands and receptors with high confidence, hits in the “scaffold” level species were sporadic. Further evidence for TGF-β signaling divergence in *H. polygyrus* is that the *H. contortus* genome contains two type I and a type II TGF-β receptor that clearly match those receptors in *C. elegans*, including their ligand binding domains. Given this conservation, *H. contortus* may not need TGM proteins to suppress the host immune response. However, we had no other choice but to focus on *H. contortus* in our search for TGM proteins outside *H. polygyrus*.

**Fig. 8.**
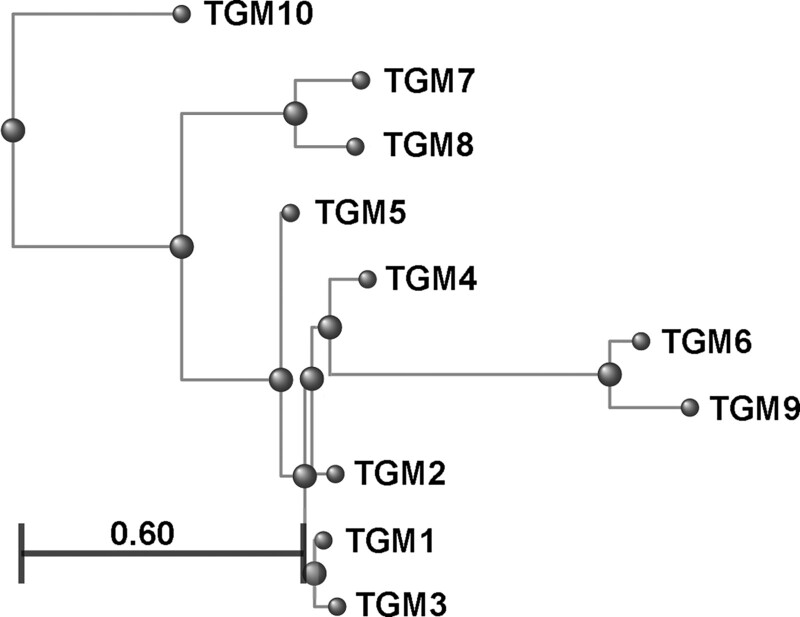
Minimum evolution tree of the ten *H. polygyrus* TGM proteins. All TGM family members were retrieved via BLASTp with TGM1 against *H. polygyrus*. Sequences were aligned with COBALT (ncbi.nlm.nih.gov/tools/cobalt/cobalt.cgi?CMD=Web) and then an unrooted minimum evolution tree was generated (ncbi.nlm.nih.gov/blast/treeview/treeView). Branches are drawn to scale with regard to the number of substitutions per site. The scale bar demonstrates that the difference between two family members can be as high as 60% of their amino acids. Note that a minimum evolution tree depicts the set of relationships with the smallest total sum of branch lengths. As a result, tree nodes have no associated statistics. The most divergent member is TGM10 on a branch of its own at the top. TGM10 was unaffected by the numerous duplications creating the other family members and thus may approximate the ancestral sequence.

For an effective search, we should employ the oldest of the ten *H. polygyrus* TGM proteins. To identify the oldest protein, we created an unrooted minimum evolution tree (fig. 8). TGM10 appeared as the most divergent family member, on a branch by itself from the node at the far left. Plus, the fact that TGM10 contains only three (D1/2/5) of the five CCP domains of TGM1 suggested TGM10 may approximate the ancestral TGM protein. A corollary is that the duplications creating D1/2/5 are older than the duplications creating D3/4.

**Fig. 9.**
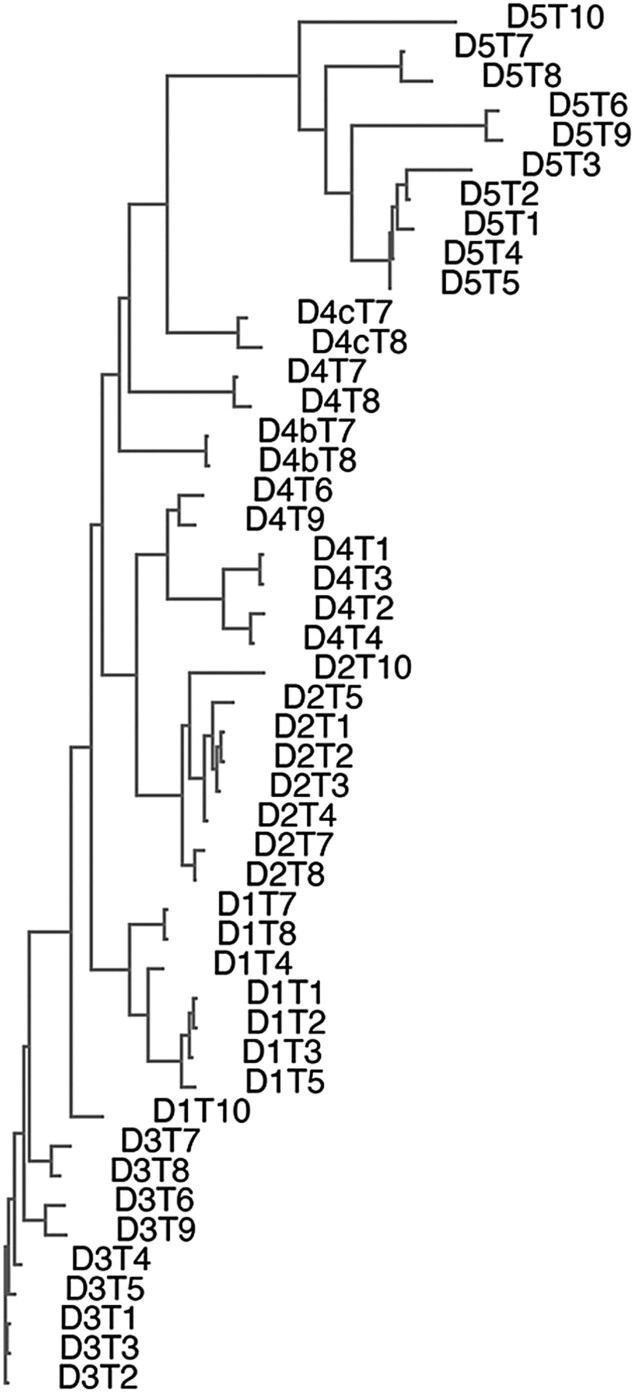
Neighbor-joining tree of all domains from the ten *H. polygyrus* TGM proteins. Domain number is indicated by the prefix D, and TGM protein number is indicated by the prefix T. After alignment in Clustal Omega (fig. 5), an unrooted neighbor-joining tree was generated (www.ebi.ac.uk/Tools/phylogeny/simple_phylogeny/). Branches are drawn to scale with regard to the number of substitutions per site. No scale bar is shown as all branch lengths are relative to their nearest neighbor. As a result, tree nodes have no associated statistics. The most divergent cluster is D5 at the top, with that cluster located on the longest branch. This indicates that domain 5 sequences have been unaffected by domain duplications and thus may approximate the ancestral domain. Within the D5 cluster, D5 of TGM10 is at the top on the longest branch, further refining the potential identity of the original TGM domain.

A search of *H. contortus* with full-length TGM10 utilizing BLASTp and tBLASTn at reduced stringency did not return any matches, nor did queries with full length sequences of the other TGMs. To identify an individual TGM10 domain for a query, we generated a neighbor-joining tree from an alignment of all domains in all TGM proteins (fig. 9). TGM10 D5 appeared as the most divergent, and it rests on the longest branch within the D5 group that resides on the longest branch of the domain groups. The fact that all domains strictly segregate from each other suggested that D5 of TGM10 may approximate the ancestral CCP module from which the TGM family was built. As a reminder, the TGF-β receptor binding domains of TGM1 are D1–D3 (Mukundan et al. 2022), whereas D4/5 of TGM1 are associated with binding an immune cell coreceptor (van Dinther et al. 2023). Thus, a corollary of the identification of D5 as possibly the oldest domain is that the ability of TGM proteins to bind immune cell coreceptors may predate the ability to bind TGF-β receptors.

Utilizing tBLASTn at reduced stringency, we obtained five matches on four chromosomes with TGM10 D5 in the Moredun *H. contortus* assembly (table 3; supplementary table S1, Supplementary Material online). Four of the five matches to TGM10 D5 show a core of identical amino acid sequences, but they are of differing lengths, suggesting that they are not assembly artifacts. Each of the 5 matches covers a minimum 49 of the 80 residues of TGM10 D5 with reasonable confidence—at least 30% amino acid identity and 50% similarity with 2 of the 4 cysteines. Chromosome 2 had two matches that were 21 Mb apart, thus not likely from the same protein. Searching similarly with the other two TGM10 domains identified a single match to D2 in the Moredun *H. contortus* genome on Chromosome 5. The D2 match is 8 Mb away from the D5 match on the same chromosome, thus not likely from the same protein. With TGM10 D2/5 hitting six independent sites, it seems that two TGM10 building blocks are present in *H. contortus*, but they are unlinked in this assembly.

**Table 3:** TGM Domain Matches (Trichostrongyloidea Divergence 186 Ma^a^)

*Heligmosomoides polygyrus* ^b^	*Haemonchus contortus* ^c^	tBLASTn^d^ % Identity (Residues)
	**Moredun Strain Reference Genome**	
TGM10 D5 (AVN88301; residue 176–251: 76 aa) All four 33% matches have identical sequence	Chromosome 1 (LS997562)	33% (61)
	Chromosome 2 (LS997563) 2 hits 21 MB apart	30% (49) 33% (57)
	Chromosome 4 (LS997565)	33% (57)
	Chromosome 5 (LS997566)	33% (57)
TGM10 D2 (AVN88301; residue 96–175: 80 aa)	Chromosome 5 (LS997566) 8 MB away from Moredun D5 hit above	39% (54)
TGM2 TGM4 TGM7 D2 AVN88293; AVN88295; AVN88298 All match to same protein	Metridin ShK toxin domain containing protein (Chromosome 2; CDJ81543) Not same hit TGM10 D2 or D5	TGM2: 22% (45)
		TGM4: 22% (63)
		TGM7: 23% (43)
	**NZ strain genome Palevich** GBE 2019	
TGM10 D5 Matches not identical to each other or to Moredun hits on Chr 2	Chromosome 2 (CP035801) 2 hits 1.6 kb apart	31% (49) 33% (57)
TGM10 D1 (AVN88301; residue 19–95: 77 aa) chr 1 hit identical Chr2	Chromosome 1 (CP035805)	33% (52)
	Chromosome 2 (CP035801) 8 MB away from NZ D5 hit above	35% (66)

a By analogy to divergence of *C. elegans* superfamily Rhabditoidea (timetree.org).

b Nematoda, Chromadorea, Strongylida, Trichostrongyloidea, and Heligmosomatidae (intestinal parasite of mouse).

c Nematoda, Chromadorea, Strongylida, Trichostrongyloidea, and Haemonchidae (intestinal parasite of ruminants).

d tBLASTn alignments shown in supplementary table S1, Supplementary Material online.

Searching the *H. contortus* genome with individual domains from the other nine TGM proteins identified a single common match for D2 of TGM2/4/7. The match is a 61 residue stretch of Chromosome 2 with a Metridin ShKT domain (canonically 35 amino acids with 6 cysteines). ShKT domains are, like CCP domains, widespread in animals. They are common in *H. contortus* (54 copies), but D2 only identified a single one. This match has modestly less confidence than the D5 matches, with roughly 20% identity and 50% similarity including three of six cysteines. As shown in figure 8, TGM2/4/7 each links independently to the second oldest node (second from left) in the TGM tree, indicating that they are not recent duplicates of each other. This node connects all TGM family members except TGM10. This observation suggests that D2 in the common ancestor of TGM1–9 incorporated an ShKT domain after the initial duplication of TGM10.

A closer look at the tBLASTn results in supplementary table S1, Supplementary Material online indicates that it is the C-terminal half (40 residues) of D2 in TGM2/4/7 proteins that is most similar to the *H. contortus* ShKT sequence. Perhaps as a result of exon modularity, the second exon (last 25 residues) of D2 has higher similarity to ShKT sequences (7/25 identities, 15/25 similarities) than to CCP sequences (6/25, 10/25 respectively; supplementary fig. S1, Supplementary Material online). Insertion of an intron into the ShKT region and loss of visibility of the ShKT region in D2 of other family members are likely due to diversification over time. Creation of the initial CCP/ShKT chimeric D2 would facilitate the neofunctionalization of TGM1-9.

This circumstance is not unique to *H. polygyrus*, as composite domains containing an ShKT fragment are present in other parasitic nematodes (Gems et al. 1995; Gems and Maizels 1996; Loukas et al. 2000). Composites are also found in mammals, for example in CRISP proteins (Gibbs et al. 2006). Taken together, the ShKT domain seems a versatile and easily transposed structural element that has been deployed multiple times in metazoan evolution.

An additional chromosome-level genome assembly of *H. contortus* is derived from a New Zealand population (Palevich et al. 2019). There is no easy way to reconcile the two genome assemblies as sequence diversity among geographic strains in *H. contortus* is considerable (Gilleard and Redman 2016). In the NZ genome, *H. polygyrus* TGM10 D5 has two hits to Chromosome 2 that are 1.6 kb apart on the same strand suggesting that they may represent two domains of the same protein. This is the only evidence of a modular TGM protein in *H. contortus*. Other observations are that TGM10 D1 has two hits in the NZ genome, although it had none in the Moredun genome. One hit is on Chromosome 1 and the other on Chromosome 2, although neither is near either TGM10 D5 match. D2 of TGM2/4/7 has no match in the NZ genome.

Taken together the two TGM trees and the *H. contortus* data support the hypotheses that TGM10 is the oldest family member, TGM10 D5 is the oldest domain, and there may not be intact TGM proteins outside *H. polygyrus*. Also supported is the hypothesis that D2 of TGM1-9 had input from an ShKT domain.

The answer to “why were TGM proteins needed for *H. polygyrus* survival” is that their own TGF-β pathway seems too divergent to modulate the host immune response. The answer to “when did the TGM family originate” is that the entire family of ten multidomain TGM proteins appears to have evolved from CCP and ShKT domains within the *H. polygyrus* lineage in response to specific selective pressure from the mouse host. The modular nature of TGM proteins allowed selection to drive different domains of the same protein in different directions. The example is TGM1 D1/2/3 targeting TGF-β receptors for immune modulation, whereas TGM1 D4/5 target immune cell-specific coreceptors to provide precision (van Dinther et al. 2023).

## Discussion

Multiple evolutionary mechanisms influenced the TGM family along its molecular path through the adaptive landscape. This family of modular proteins is found in the murine parasite *H. polygyrus*, with at least one family member able to manipulate T-cells into T-regs to suppress the host immune response. The dearth of high-quality genomes in the superfamily Trichostrongyloidea prevented us from identifying any full-length TGMs outside *H. polygyrus*, but we did identify sequences related to TGM D1, D2, and D5 in the ruminant parasite *H. contortus*.

In this family, the evolutionary mechanisms of convergence and mimicry are intertwined. Here, convergence is the mechanism by which the goal of mimicking immune suppressing TGF-β proteins was achieved. The convergent route of TGM proteins to successful functional mimicry resulted in their amino acid sequence being wholly unlike those of TGF-β family members (as the Panda's thumb is wholly unlike that of primates). As a result of sequence dissimilarity, TGM proteins activate host TGF-β signaling via a distinct mechanism. TGMs need no prodomain processing, no release from latency, nor dimerization. They are fully functional as monomers that bind via distinct domains to type I and II receptors, in some cases with higher affinity than TGF-β itself. Once bound, TGMs induce the expression of TGF-β target genes. In T-cells, TGF-β signaling transforms them into T-regs with their attendant immunosuppressive functions. Functional convergence resulted from selection on the parasite to suppress the host immune response, with mimicry of TGF-β the solution.

Multiple mechanisms of molecular evolution are blended with organismal mechanisms in shaping the TGM family. Exon modularity is illustrated by several features. First, each of the five functional domains is composed of two exons each containing two cysteines. The repetitive genomic organization of TGM domains suggests exon modularity via gene duplication. Second, the intradomain introns are significantly shorter than interdomain introns allowing for more efficient duplication to create new functional units. Here, the exception supports the rule. The TGM1 D1/D2 interdomain intron is very short, but it fits the cooperative function of D1/D2 in binding TβRI (Mukundan et al. 2022). Third, the detection of ShKT sequences in the second exon of D2 from TGM2/4/7 indicates that the creation of a chimera with a preexisting CCP domain contributed to the diversification of TGM1-9.

Exon modularity can also be seen as the result of convergence. In this view, modularity eliminates the need for dimerization in binding the TGF-β receptor complex. The five domains of TGM1 have sufficient amino acid similarity, including conserved positions for multiple cysteines, to be connected to each other via simple duplication. Yet, their modest amino acid divergence allows individual domains to function as distinct modules. Thus, D1/2 together bind the TGF-β type I receptor, D3 can bind the type II receptor, and D4/5 together bind the immune cell surface protein CD44, a cell type-specific coreceptor.

The mechanisms of convergence, mimicry, and exon modularity can be illustrated solely by TGM1. Subsequently, the mechanisms of new gene origination and gene family neofunctionalization influenced the expansion of TGM10 into the TGM family. Through the lens of these five mechanisms, we attempted to answer the questions: where did the first TGM come from and can we trace the origin and historical trajectory of the TGM family?

Although the five-domain TGM1 was the first to be described, our alignments and trees indicate that the three-domain TGM10 is the oldest. They also suggest that TGM10 D5 is the oldest module and that D2 of TGM1-9 had independent input. This information is consistent with the biochemical demonstration that D1/2 bind the TGF-β type I receptor and D5 contributes to binding the coreceptor CD44. Thus, TGM10 (with only D1/2/5) appears to represent an early, although incomplete, attempt to create a TGF-β mimic. That said, TGM10 must provide a benefit or it would have been lost. Perhaps, weak targeting of immune cells while partly binding the TGF-β type I receptor serves some purpose? We await biochemical analysis of TGM10 for new insights.

Tracing the relationships between the ten members can be done in two ways, parsimony can be applied to the presence or absence of domains to estimate similarity or a tree can be built from an amino acid alignment. When the two approaches agree, it generates confidence. For parsimony, there appears to be a single path from TGM10 containing D1/2/5. It begins with the gain of D3 in TGM5, followed by the gain of D4 in TGM1/2/3/4/7/8, followed by the loss of D1/2 in TGM6/9, perhaps coinciding with insertions in D3/4 of TGM 7/8 (fig. 5). The tree is consistent with the parsimony scenario except it places the five-domain TGM4 in a cluster with TGM6/9 that have no D1/2 (fig. 8). This placement suggests that their coreceptor domains (D4/5) have more in common than expected. Additional biochemical data are needed.

As to the origin of TGM10 D5, the weak similarity identified by comparison of TGM1 D3 with a CCP domain is the primary clue. The disulfide bonds among the four conserved cysteines serve as anchors for each structure's respective four β-sheets. CCP domain neofunctionalization then followed the expected path of duplication, mutation, and positive selection.

Our effort to understand why *H. polygyrus* needed TGMs for host immune suppression and did not utilize the much simpler solution of employing its own TGF-β proteins led to the discovery that this species contains only a single recognizable receptor. Further, this receptor displayed sequence characteristics of both type I and type II, but had no recognizable ligand binding domain. Thus, although circumstantial, the sole receptor sequence suggests that the mechanism of *H. polygyrus* TGF-β ligand–receptor interaction is sufficiently distinct from normal that the six identified *H. polygyrus* ligands would not be recognized by host TGF-β receptors. We propose that selective pressure for survival led to TGM10, to circumvent this incompatibility.

For evidence of TGM proteins outside *H. polygyrus*, we examined *H. contortus*, currently the only species with a “chromosome” level genome assembly in the Trichostrongyloidea superfamily. Together the two independent *H. contortus* genomes returned six hits to TGM10 D5, two hits to TGM10 D1, and a single hit to TGM10 D2. The presence of its three domains in *H. contortus* supports the idea that TGM10 is the oldest family member, but the disparate locations of the identified domains suggest that there may not be an intact TGM10 protein in *H. contortus*. A systematic analysis of all domains of TGM1-9 identified an interesting distinction from TGM10. D2 of TGM2/4/7 matched a single ShKT domain among the 54 in *H. contortus*. This suggested that diversification of the family beyond TGM10 included the input of an ShKT domain.

The presence of TGM D1/2/5 outside *H. polygyrus* lead us to conduct pairwise sequence comparisons of its three domains. This analysis revealed 39 and 40 substitutions between D5 and D1/D2, respectively. Comparing D1 with D2 uncovered 34 substitutions. Thus, the duplication separating D1/D2 is younger than the duplication of D5 that created the original of this pair. Applying the protein substitution rate of Drosophila (Tamura et al. 2004) to the distance between D5 and D1/2 yields a divergence time of roughly 180 Ma, with the D1/D2 divergence at 153 Ma. These ages together with the presence of multiple D5 domains in *H. polygyrus* and *H. contortus* imply that TGM D5 may be as old as the Trichostrongyloidea superfamily. Studies in Drosophila have shown that TGM10 and eventually the entire TGM family could evolve within a single lineage from D5, via a mechanism of redundant duplication under continuous positive selection (VanKuren and Long 2018).

In summary, the TGM family of *H. polygyrus* came into existence via an evolutionary path less traveled. The rapid creation via selection for functional convergence of a new multigene family of molecular mimics is, to our knowledge, without parallel. The family provides a unique illustration of five evolutionary mechanisms that have not been juxtaposed previously: convergence, mimicry, exon modularity, new gene origination, and gene family neofunctionalization. We found that TGMs were necessary because the *H. polygyrus* TGF-β pathway likely would not activate host receptors and that TGM building blocks are present in the ruminant intestinal parasite *H. contortus.* These findings suggest two avenues for future investigation. First, new biochemical data to assign functions to more domains and more family members. Second, new genome sequence in the Trichostrongyloidea superfamily and the *Heligmosomoides* genus to clarify TGM family origins and expansion. Continued study of TGM proteins will increase our knowledge of TGF-β signaling, host–parasite interactions, and metazoan evolutionary mechanisms.

## Supplementary Material

evad158_Supplementary_Data

## Data Availability

The authors affirm that all data necessary for confirming the conclusions of this article are present in this article, figures, tables, and supplemental information.
