# Supplementary material for: Convergent Evolution in a Murine Intestinal Parasite Rapidly Created the TGM Family of Molecular Mimics to Suppress the Host Immune Response
Source: Genome Biol Evol. 2023 Aug 25;15(9):evad158. doi: 10.1093/gbe/evad158 (PMC10516467; doi:10.1093/gbe/evad158)
Supplement: evad158_Supplementary_Data [file evad158_supplementary_data.pdf]

Supplementary Information: 1 Table and 1 Figure

Table S1. Alignments in support of Table 3 with the same rows and columns.

Heligmosomoides polygyrus[b]	***Haemonchus contortus***[c] **Moredun strain Reference Genome**	**tBLASTn % identity (resides)**
TGM10 Domain 5<br>chr1	Score Expect Method Identities Positives Gaps Frame<br>33.1 bits(107) 0.052 Compositional matrix adjust. 20/61(33%) 28/61(45%) 3/61(4%) +3<br>Query 17 SFDYETA---EVTYSFGNDGAHYFIEGSQVTGICNGYQVPLWCQDGEWIGEVKNISCDMMN 74<br>SF Y A E T N ++I G V G+C VPL E++ +V +SC + N<br>Sbjct 33565947 SFQYLPAVR*EATVLIINAEELFYILGLAVAGLCESVNVPLTIPSLEFLIKVGIMSCLLKN 33566129	
chr2	Score Expect Method Identities Positives Gaps Frame<br>33.3 bits(108) 0.041 Compositional matrix adjust. 14/49(29%) 25/49(51%) 0/49(0%) -1<br>Query 24 EVTYSFGNDGAHYFIEGSQVTGICNGYQVPLWCQDGEWIGEVKNISCDM 72<br>E+ F H++I G + G+C VPL + E++ E+ +SC +<br>Sbjct 32572747 ELVVFFIYTKEHFYILGLAIAGLCESVNVPLTIRRLEFLIEMGIMSCSL 32572601	
chr2	Score Expect Method Identities Positives Gaps Frame<br>31.8 bits(102) 0.16 Compositional matrix adjust. 19/57(33%) 26/57(45%) 3/57(5%) -3<br>Query 17 SFDYETA---EVTYSFGNDGAHYFIEGSQVTGICNGYQVPLWCQDGEWIGEVKNISC 70<br>SF Y A E T N ++I G V G+C VPL E++ +V +SC<br>Sbjct 11532461 SFQYLPAVR*EATVPVINAEELFYILGLAVAGLCESVNVPLTIPRLEFLIKVGIMSC 11532291	
chr4	Score Expect Method Identities Positives Gaps Frame<br>31.3 bits(100) 0.23 Compositional matrix adjust. 19/57(33%) 26/57(45%) 3/57(5%) -3<br>Query 17 SFDYETA---EVTYSFGNDGAHYFIEGSQVTGICNGYQVPLWCQDGEWIGEVKNISC 70<br>SF Y A E T N ++I G V G+C VPL E++ +V +SC<br>Sbjct 6065936 SFQYLPAVR*EATVLVINAEELFYILGLAVAGLCESVNVPLTIPRLEFLIKVGIMSC 6065766	
chr5	Score Expect Method Identities Positives Gaps Frame<br>31.5 bits(101) 0.18 Compositional matrix adjust. 19/57(33%) 26/57(45%) 3/57(5%) -3<br>Query 17 SFDYETA---EVTYSFGNDGAHYFIEGSQVTGICNGYQVPLWCQDGEWIGEVKNISC 70<br>SF Y A E T N ++I G V G+C VPL E++ +V +SC<br>Sbjct 10288288 SFQYLPAVR*EATVLVINAEELFYILGLAVAGLCESVNVPLTIPRLEFLIKVGIMSC 10288118	
TGM10 Domain 2	Score Expect Method Identities Positives Gaps Frame<br>33.1 bits(107) 0.065 Compositional matrix adjust. 21/54(39%) 27/54(50%) 3/54(5%) +1<br>Query 18 KETTNSGGESFEVVQPDADGKYPELTYIRRTCNEFPTDRKLQRDIAGLCYKAEW 71<br>+ S GESF+ P G + EL Y+RR+C+ F R LQ A L A W<br>Sbjct 18913942 RSVVISLGESFKR-SPSICGIFFELLYLRRSCSIFWMIR-LQYLFALLVL-ATW 18914094	
	Metridin ShK toxin domain containing protein (Chr 2; CDJ81543)	
TGM2 Domain 2<br>Not same hit TGM10 D2 or D5<br>All D2 match to same protein	Score Expect Method Identities Positives Gaps<br>29.2 bits(92) 0.044 Compositional matrix adjust. 10/45(22%) 23/45(51%) 0/45(0%)<br>Query 37 KYPELTYIKRICKNFPTDSNVQGHIIGMCYNAEWQFSSTPTCPAS 81<br>K + ++K++CK+ P + + +G+C + W+ CP +<br>Sbjct 18 KLRDRDHLKKLCKDHPRGNCLTVKAVGLCDDPAWKNEVNQICPKA 62	
TGM4 Domain 2	Score Expect Method Identities Positives Gaps<br>28.4 bits(89) 0.32 Compositional matrix adjust. 14/63(22%) 31/63(49%) 2/63(3%)<br>Query 115 VNAGINFNITVHPDAS-GKYPELTYIKRICKNFPADSKVQGHIIGMCYNAEWRFSSTPTCPPS 176<br>+ +GI+ I H + K + ++K++CK+ P + + +G+C + W+ CP +<br>Sbjct 1 MGSGID-GIKSHIKSLFDKLRDRDHLKKLCKDHPRGNCLTVKAVGLCDDPAWKNEVNQICPKA 62	
TGM7 Domain 2	Score Expect Method Identities Positives Gaps<br>29.2 bits(92) 0.50 Compositional matrix adjust. 10/43(23%) 23/43(53%) 0/43(0%)<br>Query 133 KYPELTYIRRICKNFPANSKVRGVIVGMCYNAEWRFSSAPVCP 175<br>K + +++++CK+ P + + VG+C + W+ +CP<br>Sbjct 18 KLRDRDHLKKLCKDHPRGNCLTVKAVGLCDDPAWKNEVNQICP 60	
	**NZ strain genome Palevich GBE 2019**	
TGM10 Domain 5<br>2 matches 1.6kb apart in same frame on chr2 (could be domains of same protein); neither is identical to the TGM10 Domain 5 Moredun hits chr2	Score Expect Method Identities Positives Gaps Frame<br>34.6 bits(113) 0.014 Compositional matrix adjust. 15/49(31%) 25/49(51%) 0/49(0%) -2<br>Query 24 EVTYSFGNDGAHYFIEGSQVTGICNGYQVPLWCQDGEWIGEVKNISCDM 72<br>EV F H++I G + G+C VPL + E++ E+ +SC +<br>Sbjct 50823763 EVVVFFIYTKEHFYILGLAIAGLCESVNVPLTIRRLEFLIEIGIMSCSV 50823617<br>Score Expect Method Identities Positives Gaps Frame<br>32.8 bits(106) 0.061 Compositional matrix adjust. 15/49(31%) 23/49(46%) 0/49(0%) -2<br>Query 24 EVTYSFGNDGAHYFIEGSQVTGICNGYQVPLWCQDGEWIGEVKNISCDM 72<br>EV F H++I G + G+C VPL E++ E+ SC +<br>Sbjct 50821981 EVVVFFIYTKDHFYILGLAIAGLCESMNVPLTITRLEFLIEIGITSCSL 50821835	
TGM10 Domain 1<br>chr2 hit is 8 MB away from pair of Domain 5 hits on chr2 above	Score Expect Method Identities Positives Gaps Frame<br>31.3 bits(100) 0.25 Compositional matrix adjust. 17/52(33%) 28/52(53%) 5/52(9%) -2<br>Query 27 PTEKDSSGRYPHGTHAKRFCKGSDEEAGLFVAICVKYRW--VYYKDVKPCPD 76<br>P+ + +GR T + KG DEE +F+ + Y+W ++ DV+P PD<br>Sbjct 3498668 PSGNNGNGRE---TLLFQ*GKGQDEEGVFFLLVVNNYKWLSLFRSDVQPPPD 3498522<br>Score Expect Method Identities Positives Gaps Frame<br>37.5 bits(124) 0.002 Compositional matrix adjust. 23/66(35%) 32/66(48%) 1/66(1%) -1<br>Query 1 GCLPLSEETATYEYY-AYSGSRYVDGNPTEKDSSGRYPHGTHAKRFCKGSDEEAGLFVAICVKYRW 65<br>G L + ++ +Y SG R +D PT K R P T K + SD+ AGL+ I V+YR<br>Sbjct 58330055 GLLSILLDSVLLQYLITVSGLRSIDTQPTIKHGESR*PCVTCCKNTMQPSDQAAGLLTTIAVRYRI 5832985	

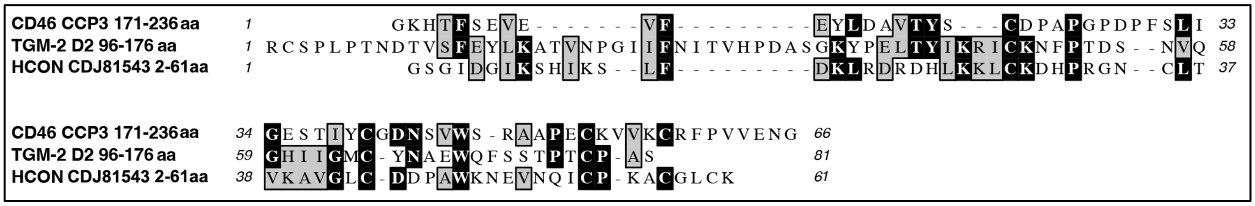

Fig. S1. ShKT domain matches the C-terminal half of TGM D2 better than a CCP domain. Alignment of TGM2 D2 (middle row) with the Metridin ShKT domain protein from *H. contortus* (previously noted in **Table 3** and **Table S1**; bottom row) and CCP D3 the from human cell surface protein CD46 (previously noted in **Fig 4**; top row). The alignment break at TGM D2 amin acid 58 marks the intra-domain intron between exons IV and V (as shown for TGM1 in **Fig. 3**). TGM2 increased similarity to ShKT begins with the basic residues upstream of the second cysteine (residue 44) and continues until the proline after the fourth cysteine (residue 79).
